# Design and Analysis of a Magnetically Coupled Multi-Frequency Hybrid Energy Harvester

**DOI:** 10.3390/s19143203

**Published:** 2019-07-20

**Authors:** Zhenlong Xu, Hong Yang, Hao Zhang, Huawei Ci, Maoying Zhou, Wen Wang, Aihua Meng

**Affiliations:** 1 School of Mechanical Engineering, Hangzhou Dianzi University, Hangzhou 310018, China; 2 School of Medicine, Zhejiang University, Hangzhou 310058, China; 3 Dalian Huarui Heavy Industry Group Co., Ltd., Dalian 116031, China; 4 CRRC Changchun Railway Vehicles Co., Ltd., Changchun 130062, China

**Keywords:** hybrid energy harvester, magnetically coupled, multi-frequency, electromechanical, broadband

## Abstract

The approach to improve the output power of piezoelectric energy harvester is one of the current research hotspots. In the case where some sources have two or more discrete vibration frequencies, this paper proposed three types of magnetically coupled multi-frequency hybrid energy harvesters (MHEHs) to capture vibration energy composed of two discrete frequencies. Electromechanical coupling models were established to analyze the magnetic forces, and to evaluate the power generation characteristics, which were verified by the experimental test. The optimal structure was selected through the comparison. With 2 m/s^2^ excitation acceleration, the optimal peak output power was 2.96 mW at 23.6 Hz and 4.76 mW at 32.8 Hz, respectively. The superiority of hybrid energy harvesting mechanism was demonstrated. The influences of initial center-to-center distances between two magnets and length of cantilever beam on output power were also studied. At last, the frequency sweep test was conducted. Both theoretical and experimental analyses indicated that the proposed MHEH produced more electric power over a larger operating bandwidth.

## 1. Introduction

In recent years, wireless sensor networks and wearable electronic devices have become widely used and vibration energy harvesters have been proposed to convert ambient vibration energy into electrical energy and serve as alternative power sources. The commonly used transduction mechanisms include piezoelectricity [1,2,3,4], electromagnetism [5], electrostatics [6], and magnetostriction [7]. Among them, the piezoelectric energy harvester (PEH) has drawn more and more research attention, due to its high energy density, easy fabrication, simple configuration, and so on.

In order to enhance energy conversion performance and environmental adaptability, researchers pay much attention to broadband piezoelectric energy harvesting. However, most of the research only focuses on a limited frequency range around a given excitation frequency. Actually, there are some vibration sources that contain two or more well-separated frequency ranges. For example, the vibration frequency of a bus floor is 111 Hz while idling, but 10.8 Hz when running at moderate speed. Laptops exhibit two vibration frequencies of 43.2 and 90.2 Hz [8]. Therefore, it is necessary to study multi-frequency energy harvesters.

There are three approaches to realize multi-frequency piezoelectric energy harvesting. (1) Arrays of energy harvesters [9,10,11,12] are generally composed of several linear single degree-of-freedom (DOF) piezoelectric energy harvesters with different resonant frequencies. They vibrate independently with respect to each other. However, this approach requires complex interface circuits. In addition, the energy conversion efficiency is low, because only one generator resonates at a certain frequency. (2) Multiple DOF energy harvesters [13,14,15,16,17,18] capture vibration energy through the multiple modes of the system, especially by their first two modes. The output power is therefore easily optimized when these resonant frequencies fit the frequencies of the vibration source. However, once the multiple DOF structure is fabricated, its frequencies are hardly changed. Furthermore, the linear multiple DOF oscillators generally have narrow operating bandwidth. (3) Coupled oscillators’ structure [19,20,21,22] usually denotes the same generating elements coupled by magnetic interaction. This magnetic interaction contributes to exciting high-energy oscillations of oscillators and improves the output power. What is more, the distribution of resonant frequencies can be tuned by changing the distance between magnets. It should be noted that piezoelectric oscillators are not good at collecting the low-frequency (<30 Hz) vibration energy, especially for micro-scale structures. Considering these prototypes and reasons, a reasonable solution is to couple the piezoelectric oscillator with one or more spring oscillators [23,24], which improves capturing the low-frequency vibration energy and increases the output power. Hence, Xu et al. [25] proposed a novel tunable multi-frequency hybrid vibration energy harvester using piezoelectric and electromagnetic conversion mechanisms, which was more conducive to improving efficiency in the low-frequency range.

As for the vibration sources, like laptops with more high-frequency vibration, three magnetically coupled multi-frequency hybrid energy harvesters were designed, modeled, experimentally validated, and compared in this paper. The generating characteristics of the optimized structure were studied further.

## 2. Design

**Figure 1:**
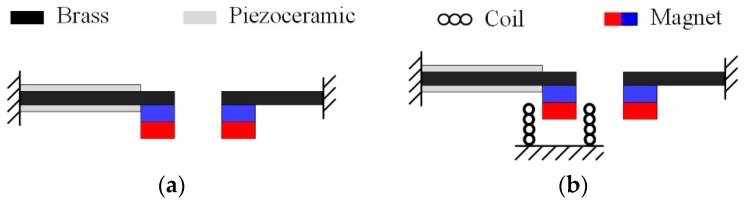

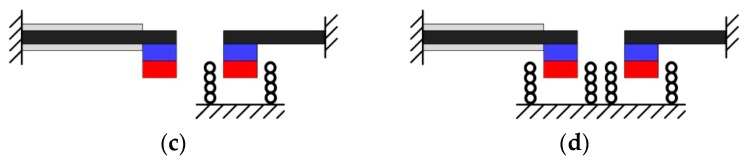
Schematic diagram of various multi-frequency vibration energy harvesters: (**a**) multi-frequency piezoelectric energy harvester (MPEH); (**b**) Pc-M type MHEH; (**c**) P-Mc type MHEH; (**d**) Pc-Mc type MHEH. Note that the characters P, c, and M denote piezoelectric oscillator, induction coil, and magnetic oscillator, respectively.

Figure 1a represents the typical nonlinear PEH with magnetically coupled dual beams, as reported [24] previously. It consists of a piezoelectric oscillator and a magnetic oscillator. Due to the property of piezoelectric material, the piezoelectric oscillator is more suitable for working in the high-frequency range than magnetic oscillator. Therefore, the magnetic oscillator is used to enhance the vibration of the piezoelectric one through magnetic interaction under low-frequency excitation, resulting in an increase of output power. Assume that the resonant frequencies of piezoelectric and magnetic oscillators are respectively matched to two discrete frequencies of vibration source, and then this nonlinear PEH can be called multi-frequency PEH (MPEH). When the excitation force is exerted on the MPEH, the magnets interact with each other and induce a varying magnetic field around it. If an induction coil is placed around the magnets, electrical power can be generated simultaneously from the piezoelectric layers and induction coil. Therefore, we propose three types of magnetically coupled multi-frequency hybrid energy harvesters (MHEHs), which are respectively named Pc-M, P-Mc, and Pc-Mc, as shown in Figure 1. Note that the characters P, c, and M denote piezoelectric oscillator, induction coil, and magnetic oscillator, respectively. To our knowledge, there has been no such design reported before.

## 3. Modeling

Considering that the MHEH is excited by low-frequency, small-amplitude vibration sources, the following assumptions are made: (1) magnets are regarded as rigid mass points with no moment of inertia; (2) the substrate and the piezoelectric layers are well attached to ignore the influence of the adhesive layer; (3) the cantilever beam is regarded as a Euler–Bernoulli beam, regardless of the moment of inertia and shear deformation; (4) the electric field in the piezoelectric layers is uniformly distributed along the thickness direction (*z*-axis direction); (5) two magnets vibrate along the vertical direction with no horizontal displacements, which are also axially aligned with the induction coils located in the bottom. The piezoelectric layers work at d_31_ mode. As shown in Figure 1, two magnets have the same magnetization directions.

### 3.1. Magnetic Force

**Figure 2:**
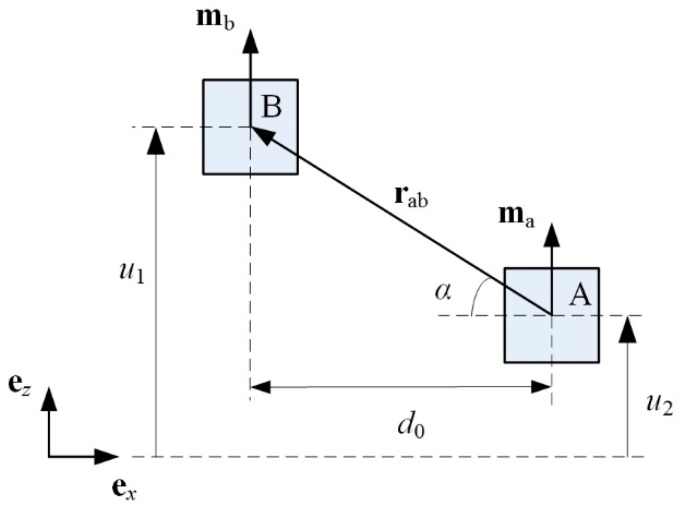
Schematic diagram of the relative positions of two magnets.

The magnetic interaction changes the equivalent spring stiffnesses of the piezoelectric and magnetic oscillators. As a result, the resonant frequency and generating performance of the MHEH can be tuned by varying the separation distance between two magnets. Magnetic force between two magnets can be calculated based on the dipole–dipole model [25]. The two magnets have the same geometrical dimensions. As illustrated in Figure 2, the magnetic moment vectors of magnets A and B, respectively are **m**_a_ and **m**_b_. The initial separation distance between two magnetic dipoles is *d*_0_. At a given time, the vertical displacements of magnet A and B are *u*_2_ and *u*_1_, respectively. The distance vector between two dipoles is **r**_ab_.

The magnetic field generated by magnet A at the location of magnet B is given by

(1) $$B_{\mathrm{ab}}=-\frac{\mu_{0}}{4\pi }\nabla \frac{m_{a}r_{\mathrm{ab}}}{r_{\mathrm{ab}}^{3}}=-\frac{\mu_{0}}{4\pi }\left[\frac{m_{a}}{r_{\mathrm{ab}}^{3}}-(m_{a}\cdot r_{\mathrm{ab}})\cdot \frac{3r_{\mathrm{ab}}}{r_{\mathrm{ab}}^{5}}\right]$$

where *μ*_0_ = 4*π* × 10^−7^ H/m is the permeability of vacuum.

The magnetic potential energy of magnet B is

(2) $$U_{m}=-B_{\mathrm{ab}}\cdot m_{b}=\frac{\mu_{0}m_{a}m_{b}}{4\pi r_{\mathrm{ab}}^{3}}\left[1-3\frac{u_{0}^{2}}{r_{\mathrm{ab}}^{2}}\right]$$

where *u*_0_ = *u*_1_ − *u*_2_.

The magnetic force exerted by magnet A on magnet B can be calculated as

(3) $$F_{m}=\frac{3\mu_{0}m_{a}m_{b}}{4\pi r_{\mathrm{ab}}^{4}}\left[{\hat{r}}_{\mathrm{ab}}+\sin\alpha {\hat{m}}_{a}+\sin\alpha {\hat{m}}_{b}-5\sin^{2}\alpha {\hat{r}}_{\mathrm{ab}}\right]$$

where ${\hat{r}}_{\mathrm{ab}}$, ${\hat{m}}_{a}$, and ${\hat{m}}_{b}$ are the unit vectors, and *α* is the angle.

The vertical and horizontal components of **F**_m_ can be respectively expressed as

(4) $$F_{mz}=\frac{3\mu_{0}m_{a}m_{b}}{4\pi }\left[3u_{0}{\left(u_{0}^{2}+d_{0}^{2}\right)}^{-5/2}-5u_{0}^{3}{\left(u_{0}^{2}+d_{0}^{2}\right)}^{-7/2}\right]$$

(5) $$F_{mx}=\frac{3\mu_{0}m_{a}m_{b}}{4\pi }\left[-d_{0}{\left(u_{0}^{2}+d_{0}^{2}\right)}^{-5/2}+5d_{0}u_{0}^{2}{\left(u_{0}^{2}+d_{0}^{2}\right)}^{-7/2}\right]$$

The corresponding equivalent stiffnesses induced by the magnetic force components on the piezoelectric beam are given as in [26]

(6) $$K_{mz1}=-\frac{\partial F_{\mathrm{mz}}}{\partial u_{1}}=-\frac{3\mu_{0}m_{a}m_{b}}{4\pi }\left[3{\left(u_{0}^{2}+d_{0}^{2}\right)}^{-5/2}-30u_{0}^{2}{\left(u_{0}^{2}+d_{0}^{2}\right)}^{-7/2}+35u_{0}^{4}{\left(u_{0}^{2}+d_{0}^{2}\right)}^{-9/2}\right]$$

(7) $$K_{mx1}=\frac{15F_{mx}}{14L_{1}}$$

where *L*_1_ is the length of the piezoelectric beam.

Therefore, the equivalent magnetic stiffnesses applied on magnetic oscillator are obtained as

(8) $$\begin{array}{cc} K_{mz2}=K_{mz1}, & K_{mx2}=-\frac{15F_{mx}}{14L_{2}} \end{array}$$

In the case of static force balance, the displacements of two magnets meet the following condition

(9) $$K_{1}u_{1}=-K_{2}u_{2}=F_{mz}$$

where the minus sign denotes the negative direction of *z*-axis. *K*_1_ and *K*_2_ are the stiffnesses of the piezoelectric and magnetic oscillators, respectively.

The potential energy of the MHEH can then be calculated as

(10) $$U=\frac{1}{2}K_{1}u_{1}^{2}+\frac{1}{2}K_{2}u_{2}^{2}+M_{1}gu_{1}+M_{2}gu_{2}+U_{m}$$

where *M*_1_ and *M*_2_ are the equivalent masses of piezoelectric and magnetic oscillators. *g* is the gravitational acceleration.

### 3.2. Electromechanical Coupling Model

**Figure 3:**
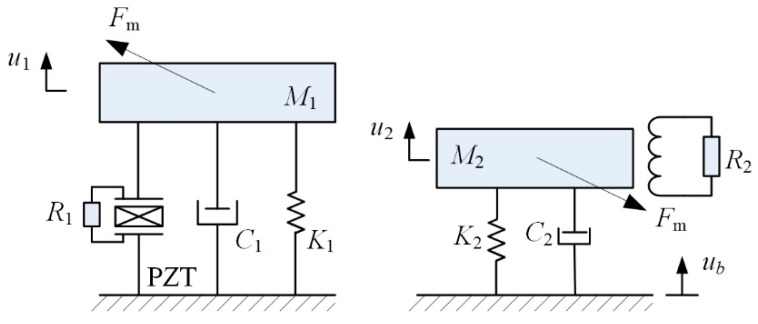
Lumped-parameter model of the MHEH.

The P-Mc type MHEH can be simplified into a lumped parameter model, as shown in Figure 3. *M*_i_, *C*_i_, and *K*_i_ (i = 1, 2) are the equivalent masses, dampings, and stiffnesses of the piezoelectric and magnetic oscillators, respectively. *θ*_p_ and *C*_p_ are the piezoelectric electromechanical coupling term and clamped capacitance of the piezoceramic layers, respectively. *θ*_e_ is the electromagnetic electromechanical coupling coefficient. *μ*_1_ and *μ*_2_ are the correction factors, respectively. *R*_1_ and *R*_2_ are load resistances connected to piezoelectric patches and induction coil, respectively. *V*_1_ is the voltage across load *R*_1_. *I*_2_ is the current in the coil. *R*_c_ and *L*_c_, respectively, are the internal resistance and inductance of the coil. *u*_b_ is the base displacement. *r*_1_ and *r*_2_ are the relative displacements of piezoelectric and magnetic oscillators to the base, respectively.

Here only the first modes of two cantilevered beams are considered. Based on the linear piezoelectric equations, Euler–Bernoulli beam theory, and Faraday’s law [27,28], the electromechanical coupling model of the MHEH subjected to harmonic excitation is expressed as

(11) $$\begin{array}{c} M_{1}{\ddot{r}}_{1}+C_{1}{\dot{r}}_{1}+(K_{1}+K_{mx1})r_{1}-\theta_{p}V_{1}-F_{mz}=-\mu_{1}M_{1}{\ddot{u}}_{b}\\ M_{2}{\ddot{r}}_{2}+C_{2}{\dot{r}}_{2}+(K_{2}-K_{mx2})r_{2}+\theta_{e}I_{2}+F_{mz}=-\mu_{2}M_{2}{\ddot{u}}_{b}\\ \theta_{p}{\dot{r}}_{1}+C_{p}{\dot{V}}_{1}+V_{1}/R_{1}=0\\ -\theta_{e}{\dot{r}}_{2}+L_{c}{\dot{I}}_{2}+(R_{c}+R_{2})I_{2}=0 \end{array}$$

Defining a state vector $X={\left[\begin{array}{cccccc} X_{1} & X_{2} & X_{3} & X_{4} & X_{5} & X_{6} \end{array}\right]}^{t}={\left[\begin{array}{cccccc} r_{1} & {\dot{r}}_{1} & r_{2} & {\dot{r}}_{2} & V_{1} & I_{2} \end{array}\right]}^{t}$, where *t* denotes the transpose operator. The Equation (11) can be written in the state space form as

(12) $$\dot{X}=\left[\begin{array}{c} {\dot{r}}_{1}\\ {\ddot{r}}_{1}\\ {\dot{r}}_{2}\\ {\ddot{r}}_{2}\\ {\dot{V}}_{1}\\ {\dot{I}}_{2} \end{array}\right]=\left[\begin{array}{c} X_{2}\\ \left[-(K_{1}+K_{mx1})X_{1}-C_{1}X_{2}+\theta_{p}X_{5}+F_{mz}\right]/M_{1}-\mu_{1}{\ddot{u}}_{b}\\ X_{4}\\ \left[-(K_{2}-K_{mx2})X_{3}-C_{2}X_{4}-\theta_{e}X_{6}-F_{mz}\right]/M_{2}-\mu_{2}{\ddot{u}}_{b}\\ -\frac{\theta_{p}}{C_{p}}X_{2}-\frac{1}{R_{1}C_{p}}X_{5}\\ \frac{\theta_{e}}{L_{c}}X_{4}-\frac{R_{c}+R_{2}}{L_{c}}X_{6} \end{array}\right]$$

The output average power delivered to the external loads *R*_1_ and *R*_2_ are, respectively, given as

(13) $$P_{p}=\frac{1}{T}\int_{0}^{T}\frac{V_{1}^{2}}{R_{1}}dt$$

(14) $$P_{e}=\frac{1}{T}\int_{0}^{T}I_{2}^{2}R_{2}dt$$

where *T* = 2*π*/*ω* is the cycle of the base excitation. *ω* is the angular velocity. Accordingly, the total output power of the P-Mc type MHEH is *P* = *P*_P_ + *P*_e_.

Similarly, the theoretical models of Pc-M type and Pc-Mc type MHEHs can be obtained as

(15) $$\begin{array}{c} M_{1}{\ddot{r}}_{1}+C_{1}{\dot{r}}_{1}+(K_{1}+K_{mx1})r_{1}-\theta_{p}V_{1}+\theta_{e1}I_{3}-F_{mz}=-\mu_{1}M_{1}{\ddot{u}}_{b}\\ M_{2}{\ddot{r}}_{2}+C_{2}{\dot{r}}_{2}+(K_{2}-K_{mx2})r_{2}+F_{mz}=-\mu_{2}M_{2}{\ddot{u}}_{b}\\ \theta_{p}{\dot{r}}_{1}+C_{p}{\dot{V}}_{1}+V_{1}/R_{1}=0\\ -\theta_{e1}{\dot{r}}_{1}+L_{c1}{\dot{I}}_{3}+(R_{c1}+R_{3})I_{3}=0 \end{array}$$

(16) $$\begin{array}{c} M_{1}{\ddot{r}}_{1}+C_{1}{\dot{r}}_{1}+(K_{1}+K_{mx1})r_{1}-\theta_{p}V_{1}+\theta_{e1}I_{3}-F_{mz}=-\mu_{1}M_{1}{\ddot{u}}_{b}\\ M_{2}{\ddot{r}}_{2}+C_{2}{\dot{r}}_{2}+(K_{2}-K_{mx2})r_{2}+\theta_{e}I_{2}+F_{mz}=-\mu_{2}M_{2}{\ddot{u}}_{b}\\ \theta_{p}{\dot{r}}_{1}+C_{p}{\dot{V}}_{1}+V_{1}/R_{1}=0\\ -\theta_{e}{\dot{r}}_{2}+L_{c}{\dot{I}}_{2}+(R_{c}+R_{2})I_{2}=0\\ -\theta_{e1}{\dot{r}}_{1}+L_{c1}{\dot{I}}_{3}+(R_{c1}+R_{3})I_{3}=0 \end{array}$$

where *θ*_e1_, *I*_3_, *R*_c1_, *R*_3_, and *L*_c1_ are the electromechanical coupling term, current, internal resistance, external resistance, and inductance of the coil located around the piezoelectric oscillator.

### 3.3. Magnetic Force and Potential Energy

As mentioned above, magnetic coupling affects the dynamic response of the MHEH. Next, we will investigate the influences of magnetic interaction on the system stiffness and potential energy. The geometric and material parameters of the MHEH listed in Table 1 are the same as those of the prototype. Here the letters A and B represent the materials from piezoelectric and magnetic oscillators, respectively.

**Figure 4:**
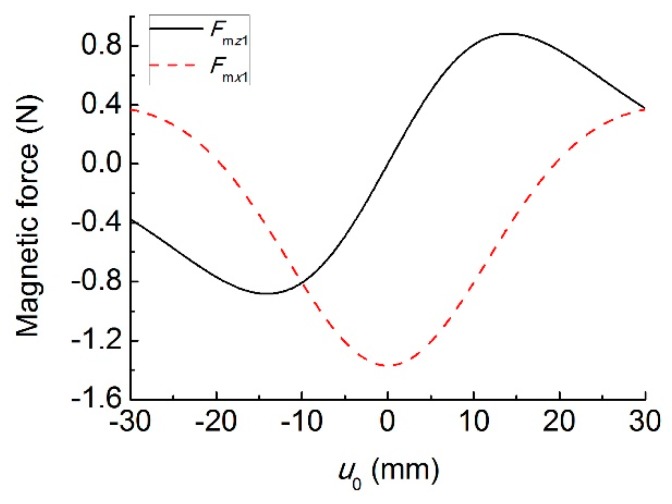
Magnetic forces versus *u*_0_.

**Figure 5:**
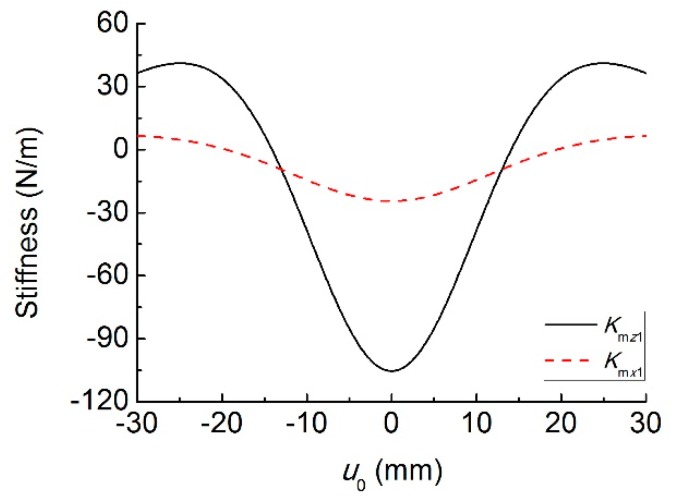
Magnetic stiffness versus *u*_0_.

Figure 4 shows the vertical and horizontal magnetic forces for different values of transverse displacement difference *u*_0_. In the range of −19.5 mm~19.5 mm, the horizontal magnetic force acts as a compressive force on the piezoelectric oscillator, while it appears as a tensile force when |*u*_0_| > 19.5 mm. The effect of the vertical magnetic force is different from the horizontal one, which acts as a repulsive force on the piezoelectric oscillator in the range of −30 mm~30 mm. Figure 5 shows the equivalent stiffnesses, *K*_m*x*1_ and *K*_m*z*1_, induced by the magnetic force for different values of transverse displacement difference *u*_0_. It can be seen that the horizontal magnetic force introduces negative stiffness to the system in the range of −19.5 mm~19.5 mm, while the range of the vertical magnetic force is −14.1 mm~14.1 mm. It means that the magnetic force is mainly repulsive when two magnets have the same magnetization direction. As a result, the equivalent stiffness and resonant frequency of the two oscillators decrease under the low-level excitation.

**Figure 6:**
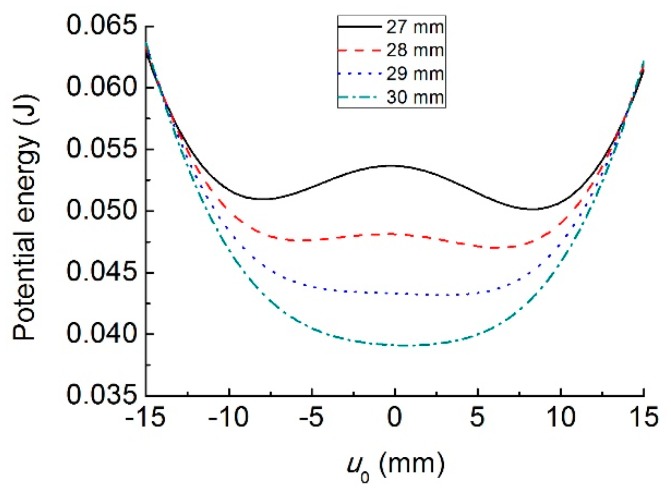
Potential energy versus *u*_0_ with different initial separation distance *d*_0_.

Figure 6 shows the relationship between the system’s potential energy and the transverse displacement difference *u*_0_ for different initial separation distance *d*_0_. With the decrease of *d*_0_, the number of the stable equilibrium point increases from one to two. Correspondingly, the system changes from monostable to bistable state. The dynamic behavior of the bistable structure is very sensitive to the system parameters and external conditions. Meanwhile, the inter-well switching can easily lead the structure to fatigue destruction. Consequently, this paper only focuses on the monostable case. Therefore, *d*_0_ is set to be larger than 30 mm.

## 4. Experimental Results and Discussion

**Figure 7:**
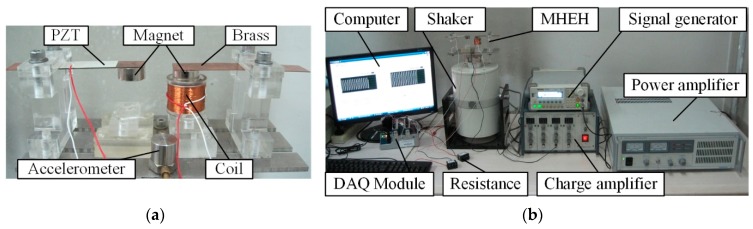
(**a**) Prototype of the P-Mc type MHEH and (**b**) experimental setup.

To verify the theoretical model, three macroscale MHEH prototypes were fabricated and an experimental setup was built up, as shown in Figure 7. The substrate of the cantilever beam was made of Phosphor Bronze (ALB Copper Alloys Co., Ltd., Xiamen, China), which was sandwiched between two P-5H piezoelectric (Baoding Hongsheng Acoustics Electron Apparatus Co., Ltd., Baoding, China) layers. Magnet material was NdFeB (N35, Ningbo Hony Technology Co., Ltd., Ningbo, China). The coil was wound with copper wire (Changzhou Wujin Enameled Wire Factory Co., Ltd., Changzhou, China). The geometric and physical properties of the prototypes are shown in Table 1. The experimental setup consisted of an electromagnetic shaker (JZK-50, Sinocera Piezotronics Inc., Yangzhou, China), a signal generator (DG-1022, Rigol Technologies Inc., Beijing, China), a power amplifier (YE5874A, Sinocera Piezotronics Inc., Yangzhou, China), a charge amplifier (CA-3, Qinhuangdao Xinheng Electronic Technology Co., Ltd., Qinhuangdao, China), and an accelerometer (YD64-310, Qinhuangdao Xinheng Electronic Technology Co., Ltd., Qinhuangdao, China). The output voltage and acceleration signal were input into a computer through the data acquisition module (NI 9229, National Instruments Co. Austin, TX, USA) and analyzed with the Labview^®^ software. Unless otherwise specified, the default value of excitation acceleration output from the electromagnetic shaker was set to be 2 m/s^2^, while the initial center-to-center distance between two magnets in the horizontal direction was 39 mm.

To compare the maximum output power generated from different MHEHs, the load resistances connected were optimized firstly. Through experimental tests, the matched load resistances connected to piezoelectric patches, coil A, and coil B were, respectively, 130 kΩ, 1500 Ω, and 2200 Ω. The fundamental resonant frequencies of the independent magnetic and piezoelectric oscillators were measured to be 27.8 Hz and 34.6 Hz, respectively.

**Figure 8:**
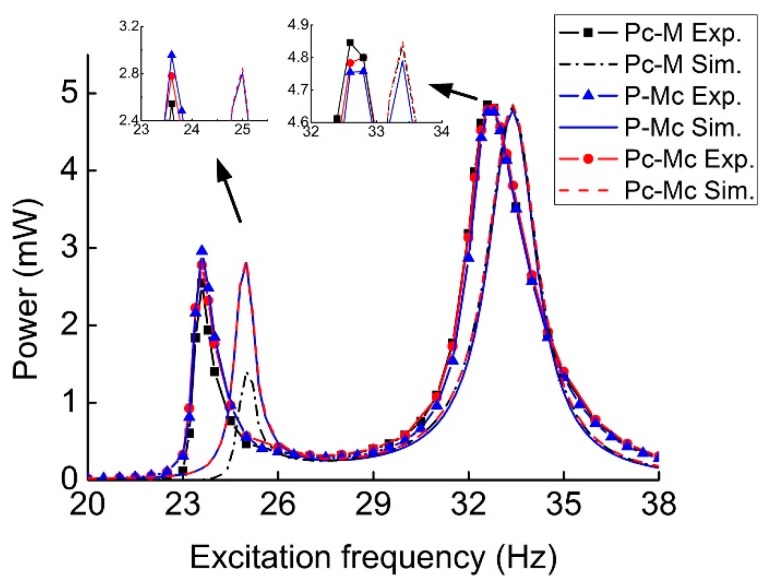
Comparison between experimental and simulation output power from three types of MHEHs.

Figure 8 illustrates the comparison of output power for three types of MHEHs. The excitation frequency was swept from 20 Hz to 38 Hz. The experimental results showed the same trends and close value as those of the simulated ones. The three peak powers at the second resonance were closer and larger than those at the first one. In regard to capturing the vibration energy with two discrete vibration frequencies, it will be beneficial to design and simplify the condition and storage circuit when the difference between two peak powers decreases. Furthermore, for most of the PEHs, the generating performance in low-frequency environment (<30 Hz) is a critical evaluation, especially for the micro-scale devices. Consequently, P-Mc type was selected as the optimal configuration, whose output power responses at the first resonance were more attractive. In the following sections, its generating performance will be further measured and analyzed.

At the first resonant frequency, the maximum electric power was generated by the P-Mc type MHEH in experimental results, while Pc-Mc type did it in simulation results. This contrast was caused by the relative motion between two magnets, which resulted in a portion of the induced electromotive force in two coils being offset. Actually, this phenomenon was not considered in the theoretical model. In the experimental results, there existed two resonant frequencies 23.6 Hz and 32.8 Hz, which corresponded to the fundamental resonant frequencies of magnetic and piezoelectric oscillators, respectively. However, the magnitudes respectively decreased by 15.1% and 5.2%. It is due to the effect of magnetic interaction, which decreased the equivalent stiffness of the oscillators. At the first resonance, P-Mc type generated the maximum output power of 2.96 mW, which was about 106.5% and 116.5% greater than that of Pc-Mc type (2.78 mW) and Pc-M type (2.54 mW), respectively. At the second resonance, Pc-M type output the maximum power of 4.85 mW, which was only 101.0% and 101.9% times that of Pc-Mc (4.80 mW) and P-Mc (4.76 mW), respectively.

Table 2 shows the comparison between two published energy harvesters and the proposed P-Mc type MHEH. They were all excited at 2 m/s^2^. Due to the difference in the geometric dimension, power density was selected as a unified metric. Although the nonlinear PEH [23] had an attractive output power and power density in the second resonant frequency f2, the gap between the first peak power and the second one was so large that it was a huge challenge for the energy harvesting circuit design. The tunable MHEH [25] reported before showed a more balanced power density than the nonlinear PEH. However, the proposed P-Mc type MHEH has the best power density.

**Figure 9:**
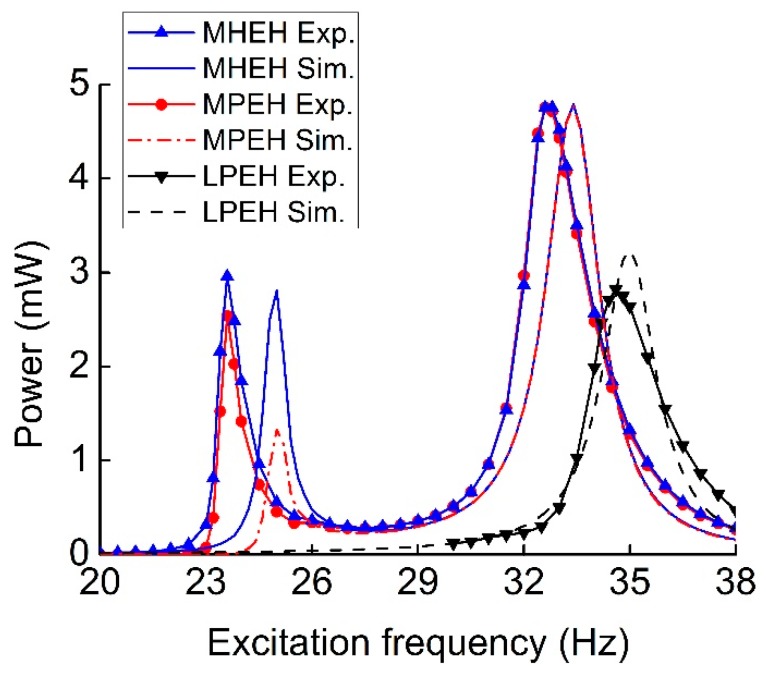
Comparison between experimental and simulation output power for the MHEH, MPEH, and LPEH.

When the induction coil and magnetic oscillator of P-Mc type were removed, a conventional linear piezoelectric energy harvester (LPEH) was developed. Figure 9 shows the comparison of output power for the MHEH, MPEH, and LPEH. Both the experimental and simulation results demonstrated the superiority of the hybrid energy harvesting mechanism. It was easy to find that there was a great discrepancy of the peak power for the MPEH at the first resonance. This was mainly because the removal of the coil reduces the damping of the magnetic oscillator. As a result, more vibration energy was converted. Nevertheless, the damping coefficient in the simulation model is always left unchanged by default.

At the first resonance of experimental results, the output power (2.96 mW) and operating bandwidth (0.91 Hz) of the MHEH respectively increased by 16.5% and 21.3%, as compared to that of the MPEH (2.54 mW and 0.75 Hz). But they had almost same bandwidth and peak output power at the second resonance. In addition, it is clear that there was a significant increment (68.8%) of the peak power generated from the MHEH at the second resonance compared to that of the LPEH (2.82 mW). The reason mainly lies in two aspects: part of the kinetic energy captured by the magnetic oscillator was delivered to the piezoelectric oscillator through the magnetic interaction, and then converted into electrical energy; the electromagnetic energy harvester (EMEH) subsystem generated electrical power simultaneously. It is worth noting that both peak powers of the MHEH and MPEH at the first resonance were close to that of the LPEH. It was demonstrated that magnetically coupled multi-frequency structure significantly improved the output power of the vibration energy harvester in the low-frequency range.

**Figure 10:**
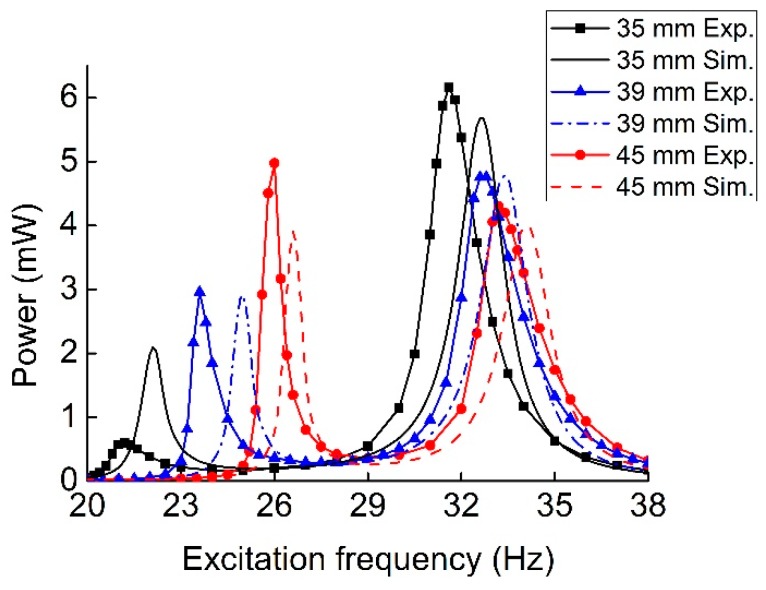
Experimental and simulation output power versus frequency for different *d*_0_.

Figure 10 shows the output power of the MHEH for different initial center-to-center distances *d*_0_ between two magnets. With the increase of the distance, the resonant frequencies gradually increased and approached the corresponding natural frequencies of two oscillators. Meanwhile, the first peak power gradually increased, whereas the second one decreased. This is because the magnetic interaction between the two oscillators became attenuated. According to the effect of magnetic distance, it needed to make a tradeoff between the resonant frequency distribution and peak output power in practical applications, so as to achieve optimal energy conversion efficiency. As shown in experimental results, it seemed that varying *d*_0_ had larger effects on the output power at the first resonance, which was not obvious in simulation results. This was also induced by the change of *d*_0_. When the distance was changed, not only the equivalent stiffness but also the damping of the MHEH varied, especially for those of the magnetic oscillator. However, the damping was set to be constant in the simulation.

**Figure 11:**
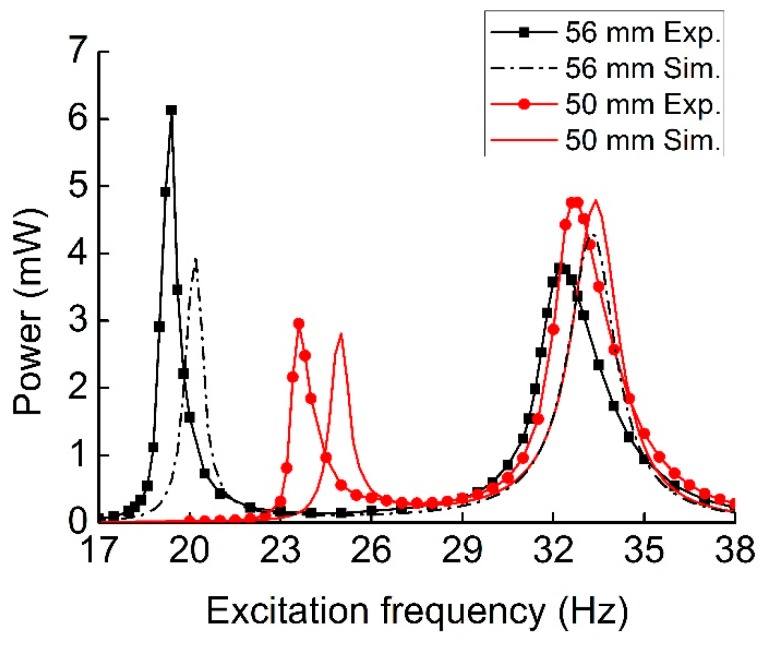
Comparison between experimental and simulation output power versus frequency for different *L*_2_.

Figure 11 shows the output power frequency response of the MHEH with different *L*_2_, where *L*_2_ is the length of the magnetic oscillator’s beam. When the length of the cantilever beam increased, the first peak output power became larger, but the corresponding resonant frequency decreased. Meanwhile, the second peak power and resonant frequency went down. The reason was that the increase of *L*_2_ led to a decrease in the equivalent stiffness *K*_2_. Therefore, the first resonant frequency induced by the magnetic oscillator went down, and its amplitude and velocity were enhanced so that the first peak power became much larger. Because the second resonance got farther away from the first one, the vibration of magnetic oscillator attenuated significantly at the second resonance, which cut down the effect of the magnetic interaction. Consequently, the amount of vibration energy harvested declined.

**Figure 12:**
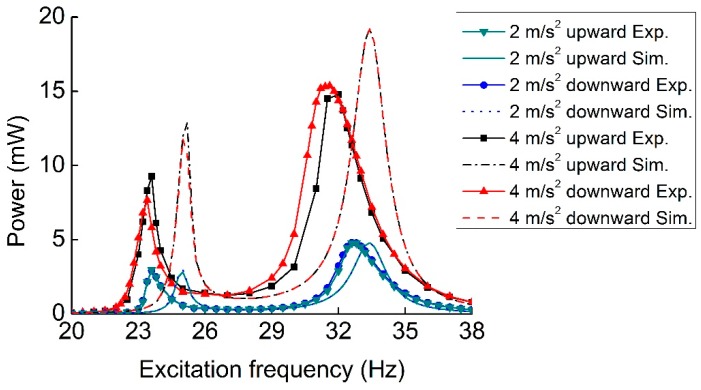
Experimental and simulation output power of the P-Mc type MHEH at different excitation accelerations in the frequency sweep tests.

Frequency sweeps can be performed to find the stable states of a hardening or softening system at high-energy and low-energy orbits. Figure 12 presents the output power of the P-Mc type MHEH for excitation frequency upward and downward sweeps under 2 m/s^2^ and 4 m/s^2^ excitation accelerations. At the low excitation level, the frequency response from upward frequency sweep was similar to that from downward frequency sweep, just like a linear system. The experimental results agreed well with the simulation results. However, as the excitation level increased, the first resonance shifted to the right and the second one shifted to the left in experimental results, accompanied by jump phenomenon. Thus, more power is generated over dual-directionally broadened bandwidth. Clearly, hardening and softening nonlinear responses occurred at the first and second resonances, respectively. The hardening case was due to the magnetic stiffness and geometric nonlinearity, while the softening case was attributed to the effect of material nonlinearities in the piezoelectric layers [29,30]. It should be noted that there were no such nonlinear responses in simulation results due to the neglect of these nonlinear factors. In the future, a more comprehensive theoretical model will be established to analyze the influences of these nonlinearities, which are not the research focus of this paper.

## 5. Conclusions

In this paper, three types of magnetically coupled multi-frequency hybrid energy harvesters were proposed, modeled, fabricated, tested, and compared, in order to optimize the performance in capturing vibration energy with two discrete frequencies. At last, P-Mc type was selected as the best configuration. Its dynamic and electric responses under harmonic excitation were analyzed through experiments and simulation. When it was subjected to the excitation with 2 m/s^2^ acceleration and the initial center-to-center distance between two magnets in the horizontal direction was 39 mm, it generated 2.96 mW and 4.76 mW at 23.6 Hz and 32.8Hz, respectively. At the first resonance, the output power and operating bandwidth respectively increased by 16.5% and 21.3%, as compared to that of the MPEH, while the second peak power was 168.8% times that of the LPEH. Both the experimental and simulation results demonstrated the superiority of the hybrid energy harvesting mechanism to the MPEH and LPEH. The resonant frequency distribution and peak output power could be adjusted by altering the center-to-center distance between two magnets. Increasing the length of the cantilever beam of magnetic oscillator could greatly improve the first peak output power. It was found that the hardening and softening nonlinear responses respectively occurred at the first and second resonances through the frequency upward and downward sweeps. In a word, the proposed magnetically coupled multi-frequency hybrid energy harvesting mechanism shows great potential in harvesting vibration energy with two discrete frequencies and it is an effective approach to improve the output power of the vibration energy harvester in the broadband low-frequency environment.

## Figures and Tables

**Table 1:** Geometric and material parameters of the MHEH.

Parameter	Value
Substrate A length × width × thickness (mm^3^)	60 × 20 × 0.5
Substrate B length × width × thickness (mm^3^)	50 × 20 × 0.54
Substrate density (kg/m^3^)	8920
Young’s modulus of substrate (GPa)	121
Piezoceramic length × width × thickness (mm^3^)	40 × 20 × 0.2
Piezoceramic density (kg/m^3^)	7386
Young’s modulus of piezoceramic (GPa)	59.77
Piezoelectric stress constant (C/m^2^)	−13.74
Dielectric permittivity (nF/m)	38.62
Magnet radius × height (mm^2^)	10 × 10
Residual magnetic flux density (T)	1.3
Magnet density (kg/m^3^)	7500
Coil turns	2000
Internal resistance of coil A (Ω)	352
Internal resistance of coil B (Ω)	360

**Table 2:** Comparison between two published energy harvesters and the P-Mc type MHEH.

Ref.	Volume (mm^3^)	Resonant Frequency f1 (Hz)	Peak Power P1 (mW)	Power Density D1 (mW/mm^3^)	Resonant Frequency f2 (Hz)	Peak Power P2 (mW)	Power Density D2 (mW/mm^3^)
[23]	945.84	22	0.013	1.37 × 10^−6^	27.7	1.658	1.75 × 10^−3^
[25]	29895	23.2	2.12	7.09 × 10^−5^	26.8	1.57	5.25 × 10^−5^
This paper	21550	23.6	2.96	1.37 × 10^−4^	32.8	4.76	2.21 × 10^−4^
